# Supplementary figures and images for: Piperlongumine as a Neuro-Protectant in Chemotherapy Induced Cognitive Impairment
Source: Int J Mol Sci. 2022 Feb 11;23(4):2008. doi: 10.3390/ijms23042008 (PMC8880369; doi:10.3390/ijms23042008)

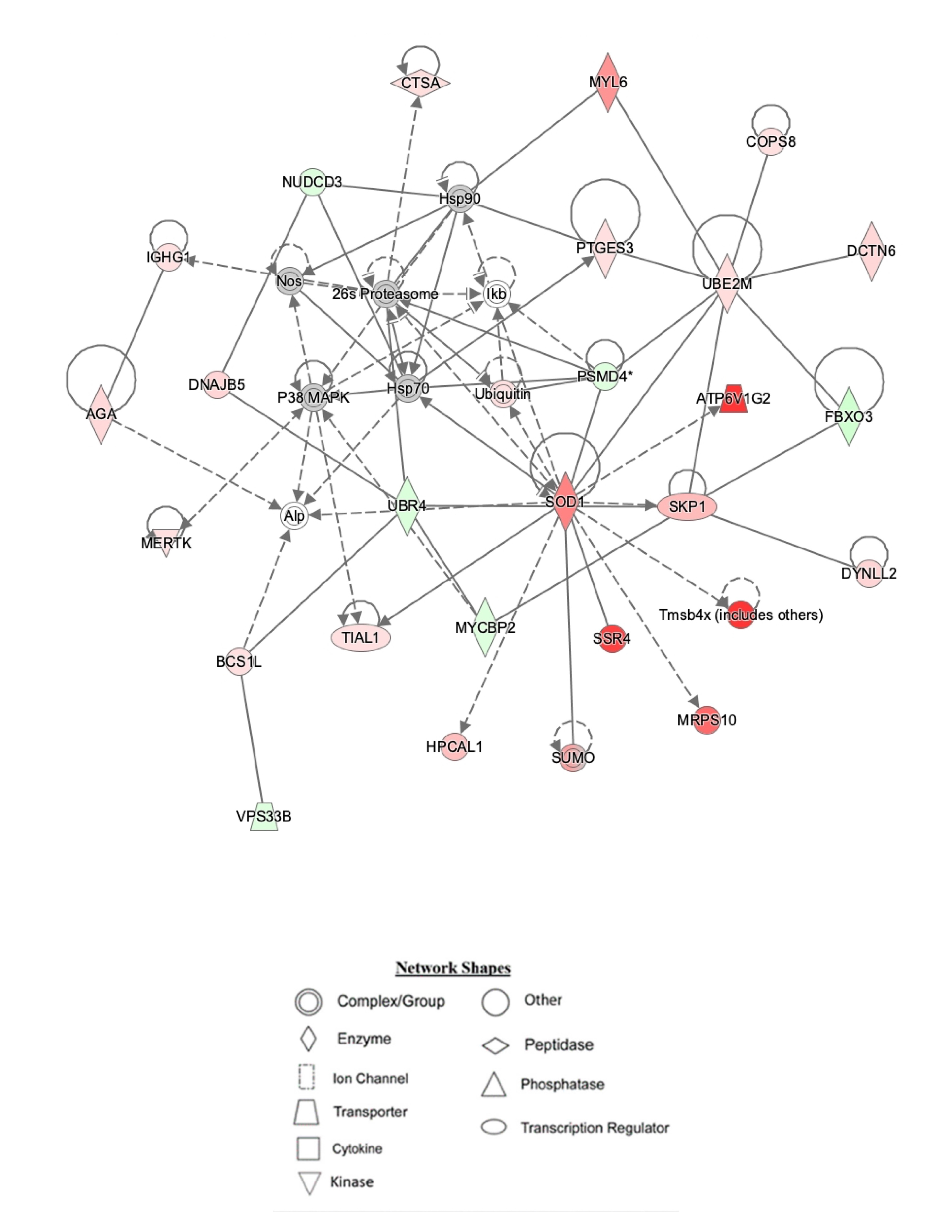

Supplement: Supplementary file 1 [file ijms-23-02008-s001.zip › Fig. S1.jpg]

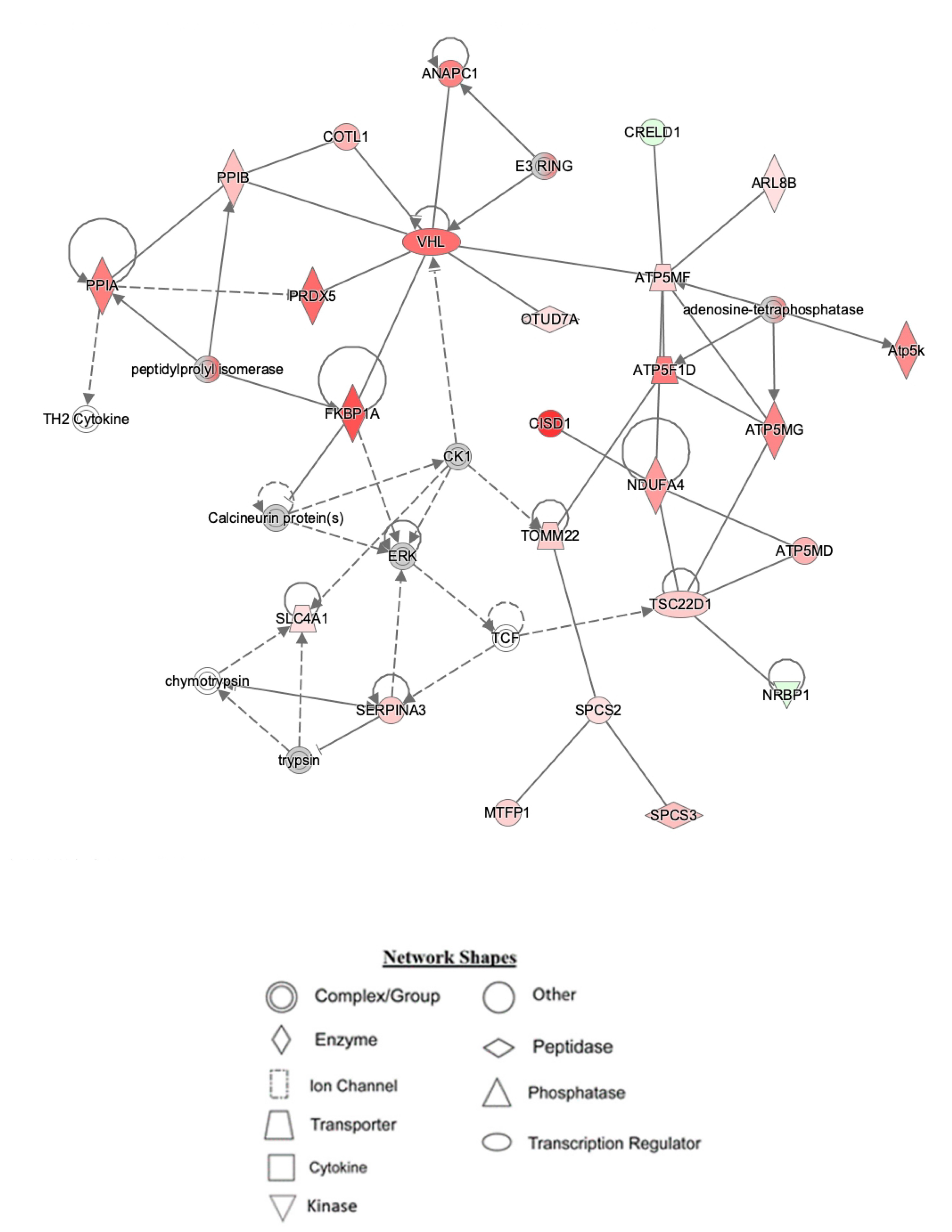

Supplement: Supplementary file 1 [file ijms-23-02008-s001.zip › Fig. S2..jpg]

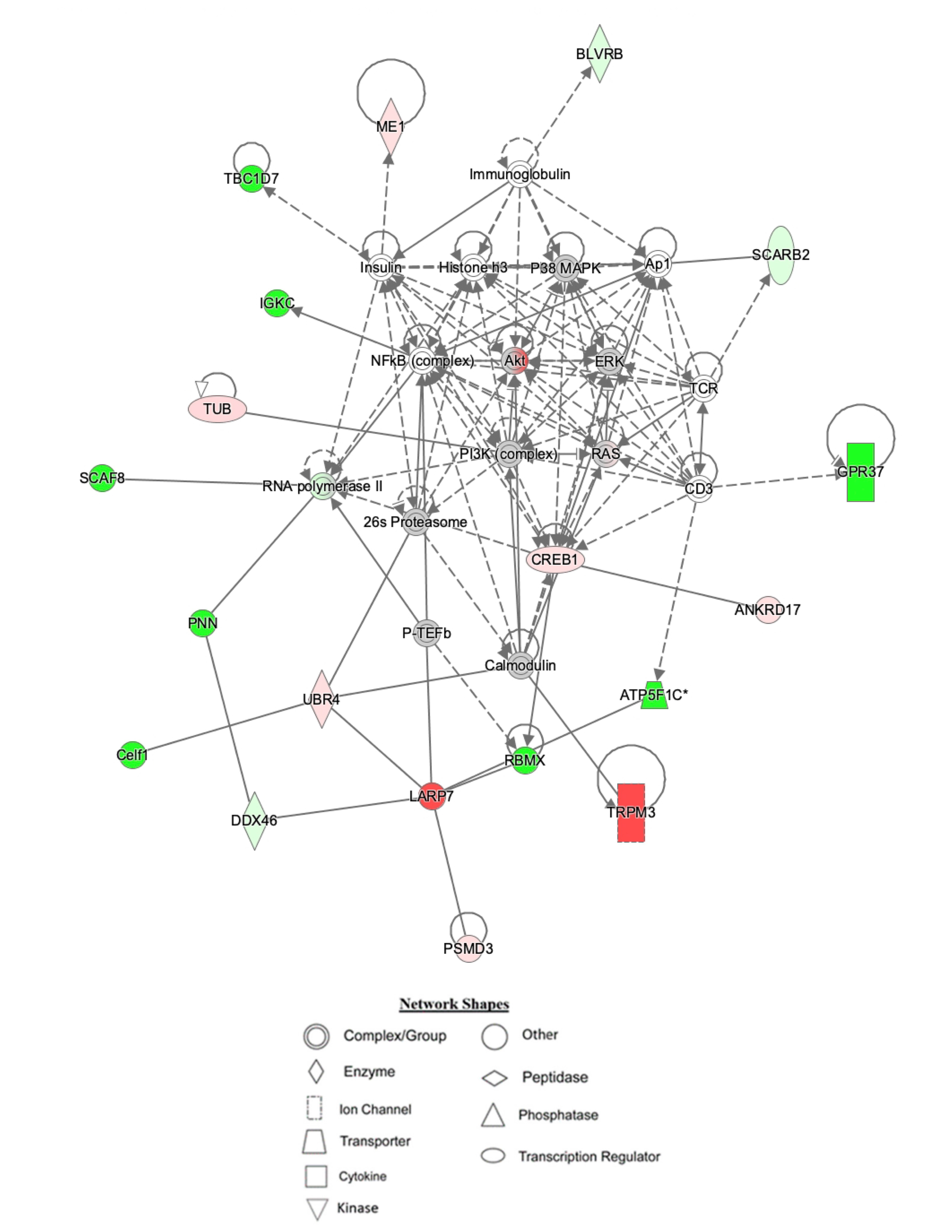

Supplement: Supplementary file 1 [file ijms-23-02008-s001.zip › Fig. S3.jpg]

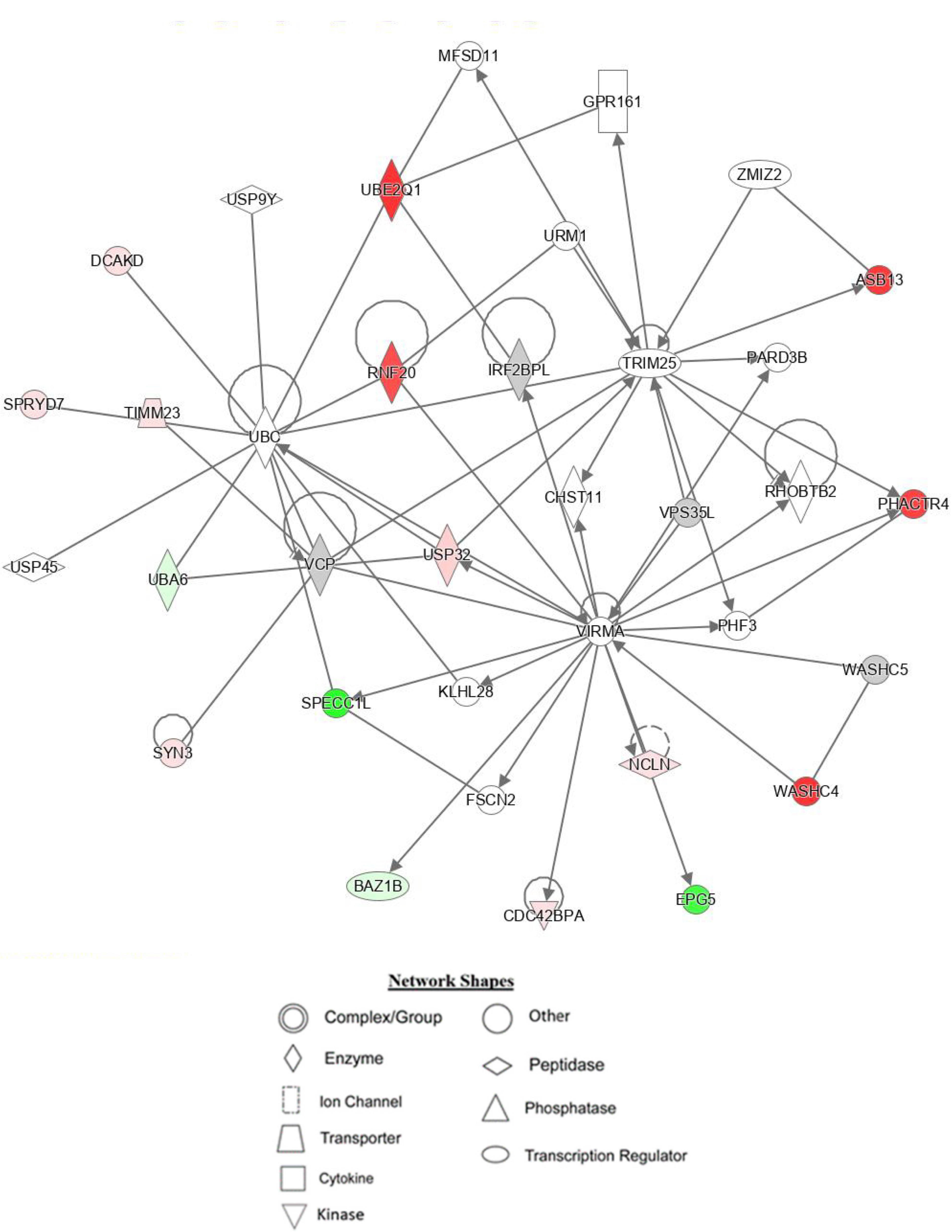

Supplement: Supplementary file 1 [file ijms-23-02008-s001.zip › Fig. S4.jpg]

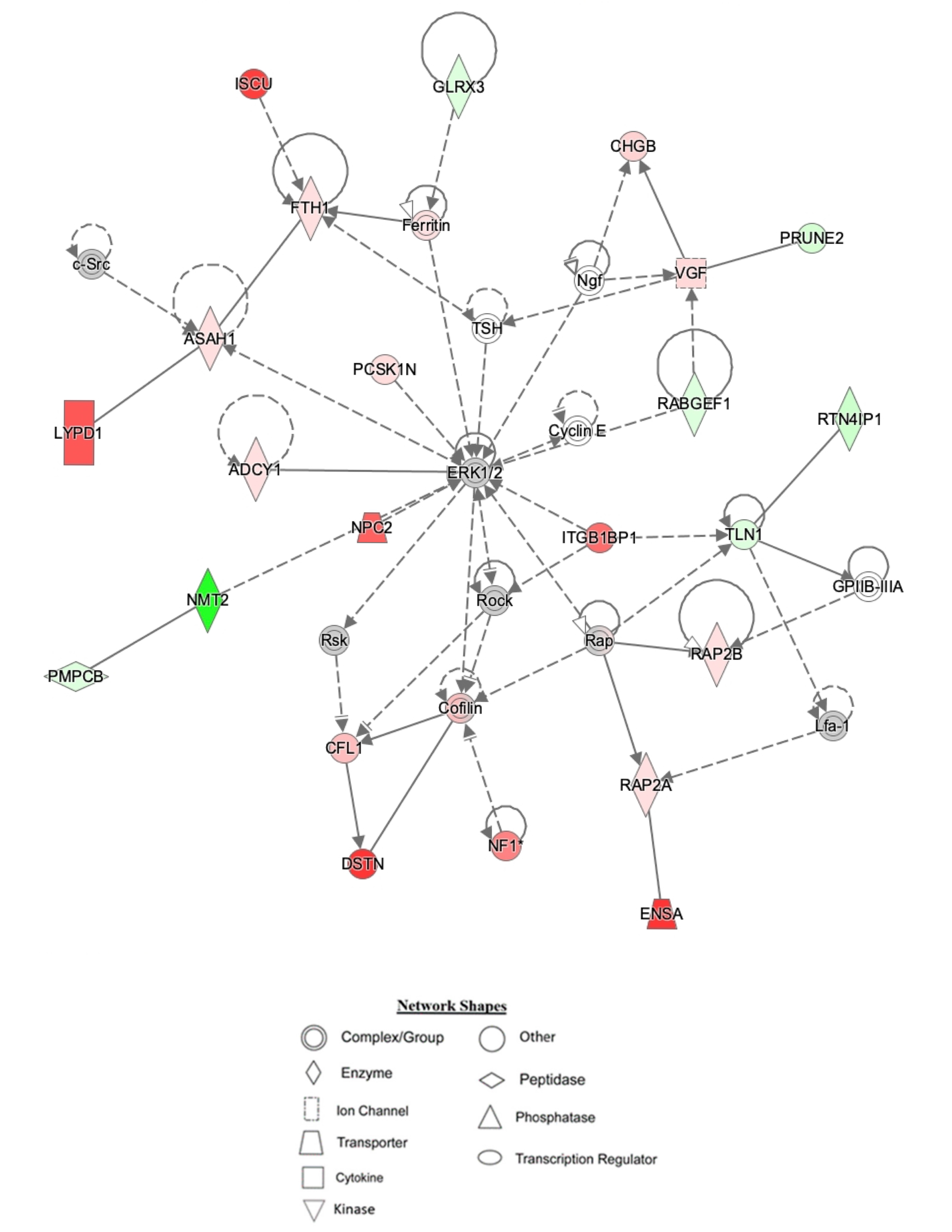

Supplement: Supplementary file 1 [file ijms-23-02008-s001.zip › Fig. S5.jpg]

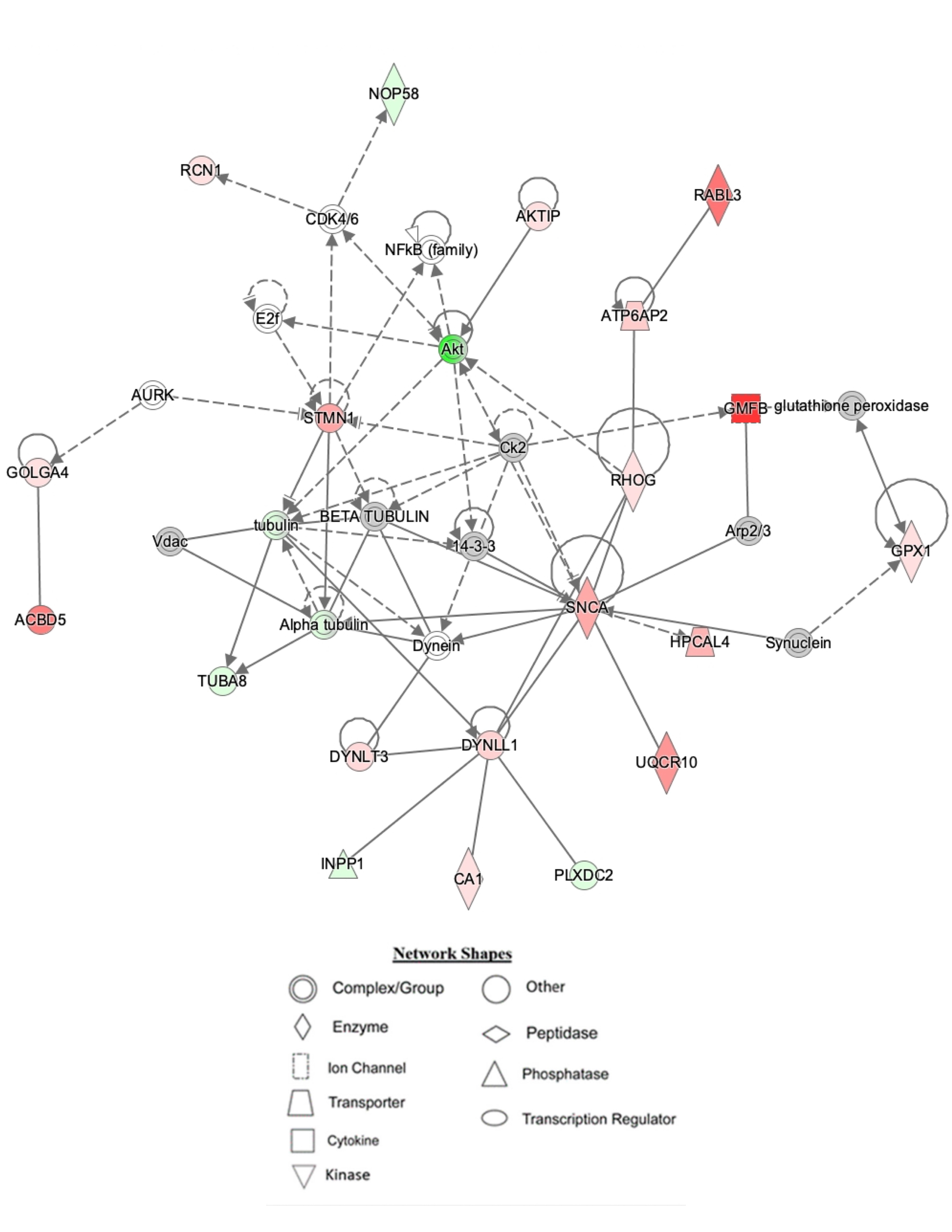

Supplement: Supplementary file 1 [file ijms-23-02008-s001.zip › Fig. S6.jpg]

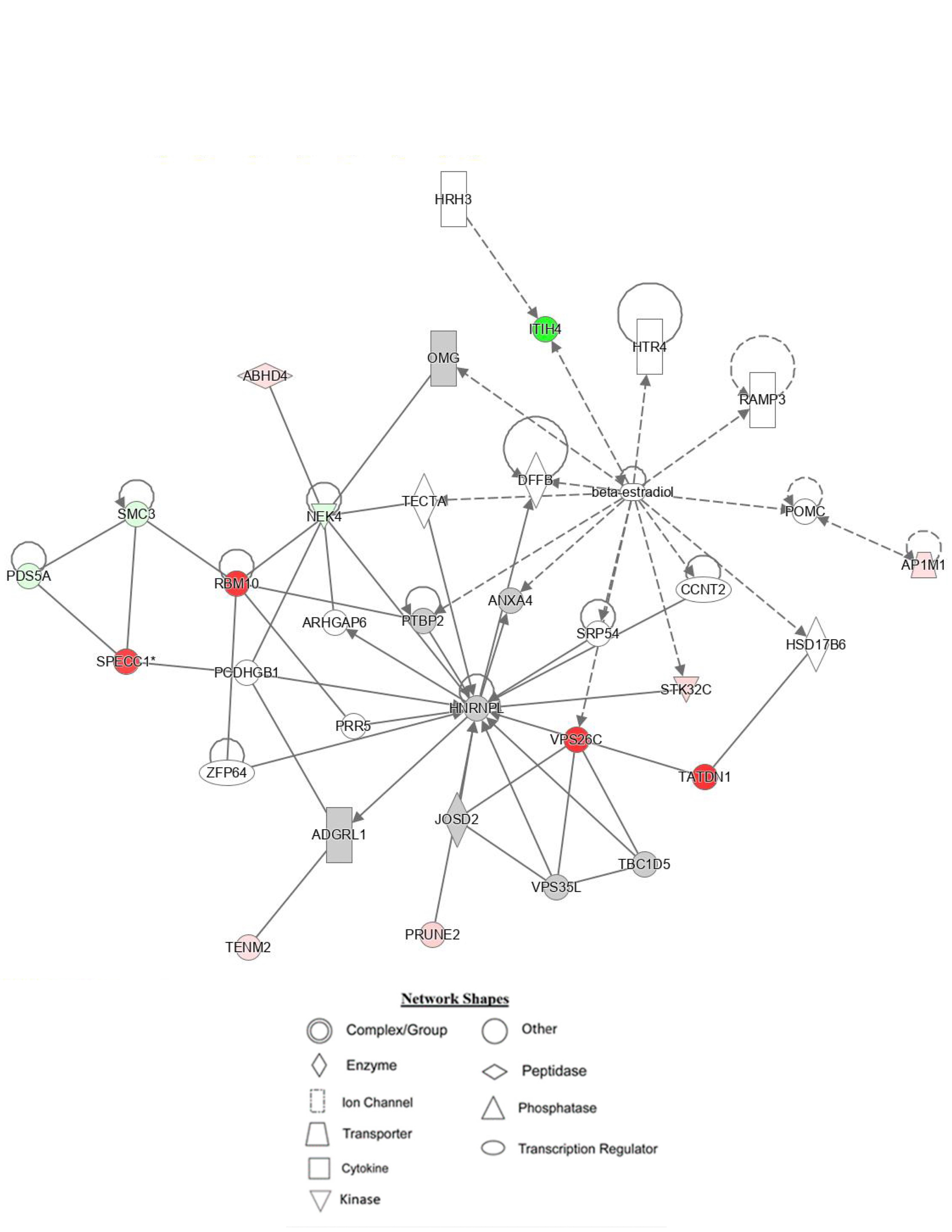

Supplement: Supplementary file 1 [file ijms-23-02008-s001.zip › Fig. S7.jpg]

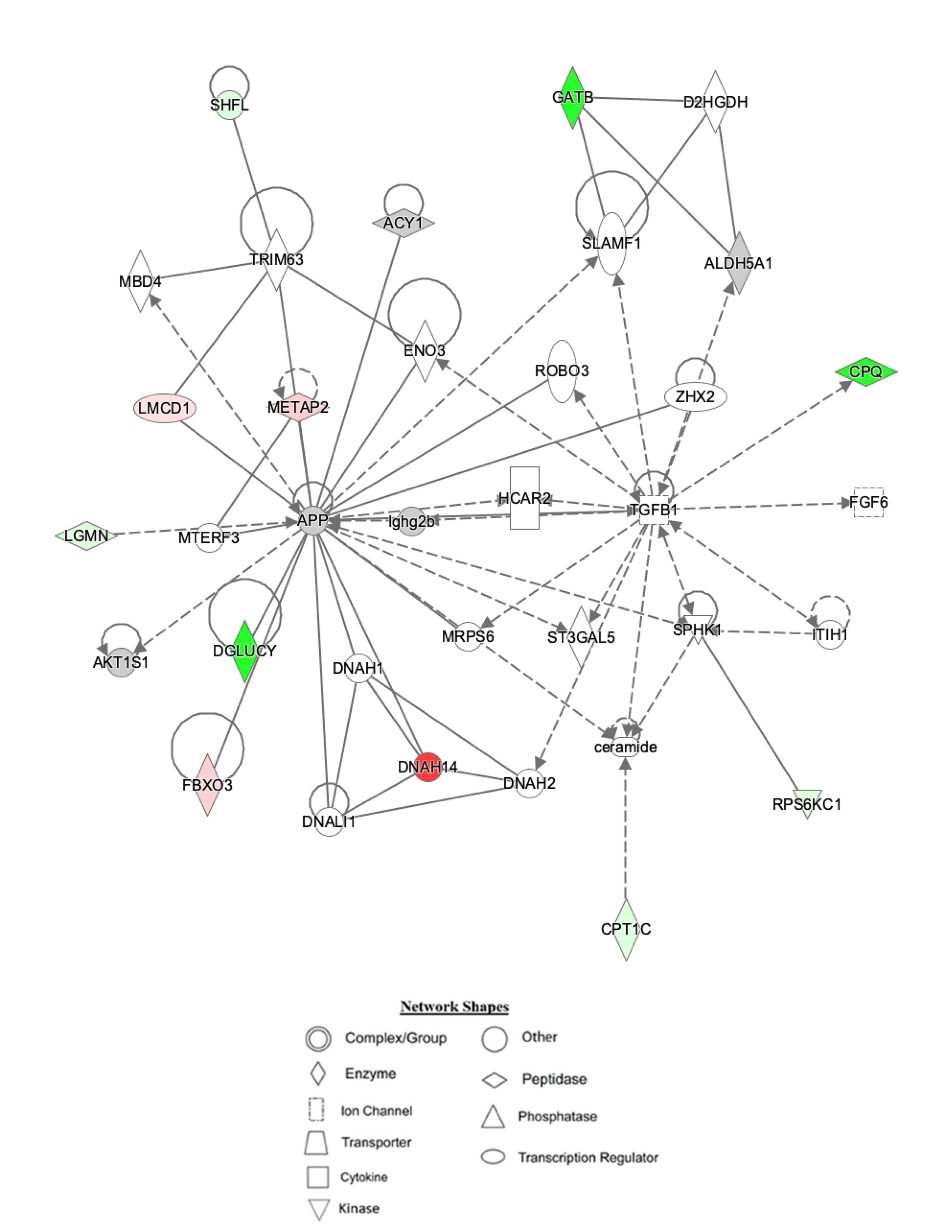

Supplement: Supplementary file 1 [file ijms-23-02008-s001.zip › Fig. S8.jpg]
